# In Patients with Coronary Artery Disease and Type 2 Diabetes, SIRT1 Expression in Circulating Mononuclear Cells Is Associated with Levels of Inflammatory Cytokines but Not with Coronary Lesions

**DOI:** 10.1155/2016/8734827

**Published:** 2016-03-31

**Authors:** Yuanmin Li, Jing Ni, Rong Guo, Weiming Li

**Affiliations:** ^1^Department of Cardiovascular Surgery, Shanghai Tenth People's Hospital, Tongji University School of Medicine, Shanghai 200072, China; ^2^Department of Cardiology, Shanghai Tenth People's Hospital, Tongji University School of Medicine, Shanghai 200072, China

## Abstract

While SIRT1 is significantly associated with atherosclerosis and diabetic complications, its relevance to coronary lesions in patients with coronary artery disease and type 2 diabetes has not been specifically investigated. Thus, we assessed SIRT1 expression in peripheral blood mononuclear cells in these patients. We found that SIRT1 expression did not significantly correlate with syntax scores from coronary angiography (*p* > 0.05). Notably, plasma levels of the inflammatory cytokines tumor necrosis factor-*α*, monocyte chemoattractant protein-1, and high-sensitivity C-reactive protein were markedly higher in diabetic patients (*p* < 0.05). In addition, SIRT1 expression was negatively correlated with levels of these cytokines, as well as that of interleukin-6 (*p* < 0.05). In summary, the data indicate that SIRT1 expression in peripheral blood mononuclear cells is significantly correlated with inflammatory cytokines levels in patients with coronary artery disease and type 2 diabetes but not with the severity of coronary lesions.

## 1. Introduction

Type 2 diabetes mellitus (T2DM) and coronary artery disease (CAD) are major public health issues in industrialized countries [1, 2]. T2DM is a major independent risk factor for CAD and accelerates the development of atherosclerosis via various mechanisms [3]. Consequently, cardiovascular complications are a major cause of mortality and morbidity in diabetic patients [4]. Indeed, patients with both diabetes and CAD are challenging to manage and comprise a growing segment of the population [5]. Therefore, therapeutic strategies for these patients are urgently needed [6, 7].

Sirtuin 1 (SIRT1), a nicotinamide adenine dinucleotide-dependent histone deacetylase that regulates glucose and lipid metabolism [8–10], is closely associated with extended lifespan due to calorie restriction [11]. Stein and colleagues [12] also found that SIRT1 prevents atherogenesis by inhibiting the formation of macrophage foam cells. Accordingly, SIRT1 expression has been reported to be significantly lower in patients with acute coronary syndrome or stable CAD [13, 14]. Finally, evidence has also accumulated to suggest that SIRT1 activation could be a therapeutic strategy to reverse atherosclerosis [15–18]. However, SIRT1 expression and its relationship with coronary lesions in patients with both CAD and T2DM have not been investigated. In this study, we tested the hypothesis that mRNA expression in peripheral blood mononuclear cells correlates with the severity of coronary lesions in diabetic patients.

## 2. Materials and Methods

### 2.1. Study Population

We screened patients with CAD who were admitted to our department between July 2013 and July 2014. In the end, we enrolled 285 consecutive patients with or without T2DM. Diagnosis of T2DM was established on the criteria from guideline [19]. These patients were 20–70 years old, had typically ischemic angina, and had undergone coronary angiography. Patients with a history of ACS < 6 months prior to enrollment were excluded, along with those who had connective tissue, autoimmune, malignant, or infectious disease within the previous month. Patients with anemia (hemoglobin < 90 g/L) and severe hepatic or renal failure or who had major operations or trauma within the previous 3 months were also excluded. Patients were treated according to routine clinical protocols and guidelines. Standard 12-lead electrocardiograms, cardiac biomarker data, plasma inflammatory biomarkers, and coronary angiographic results were collected, along with patient demographics and risk factors such as age, gender, hypertension, smoking status, and hyperlipidemia. Fifty healthy individuals were enrolled as controls. This study was approved by the local institutional ethics committee, and written informed consent was obtained.

### 2.2. Isolation of Peripheral Blood Monocytes

Blood was suspended over a layer of Ficoll reagent and centrifuged for 20 min at 1800 ×g and room temperature to separate peripheral blood mononuclear cells. These cells were then transferred to a fresh tube and washed twice with PBS. Subsequently, monocytes were isolated by magnetic-activated cell sorting (MACS), using magnetic beads coated with CD14. The purity of isolated monocytes was tested by flow cytometry as described [14], using cells stained with fluorescein-labeled CD14 antibody.

### 2.3. RNA Extraction and Quantitative Real-Time PCR

Total RNA was extracted from purified peripheral blood monocytes resuspended in Trizol reagent (Invitrogen, USA). RNA concentration, purity, and quality were assessed by optical density at 260 nm and 280 nm and by visualization of 18S and 28S rRNA. Samples (1 *μ*g) were reverse transcribed using oligo(Dt)_15_ and M-MLV Reverse Transcriptase (Promega, USA) according to the manufacturer's instructions. The resulting cDNA (2 *μ*L) was amplified by semiquantitative PCR in 25 *μ*L reactions containing 12.5 *μ*L 2x PCR-Mix, 1 *μ*L each of forward and reverse primers (10 *μ*mol/L), and sterile water. Primers were designed in Primer 5.0 to span exons to minimize amplification of genomic DNA, if present. SIRT1 primers had sense sequence 5′-CGG ATT TGA AGA ATG TTG GTT C-3′ and antisense sequence 5′-GGA-AAA-TGT-AAC-GAT-TTG-GTG-G-3′. GAPDH was amplified with sense primer 5′-ACG GAT TTG GTC GTA TTG GG-3′ and antisense primer 5′-TGA TTT TGG AGG GAT CTC GC-3′. Genes were amplified by initial denaturation at 94°C for 3 min, followed by cycles of denaturation at 94°C for 30 s, annealing for 30 s, and extension at 72°C for 50 s, and then by final extension at 72°C for 5 min. Annealing temperature and number of cycles were optimized for each gene. In particular, the number of cycles was set in preliminary trials to be within the linear range to ensure accuracy of semiquantitative analysis. PCR products (5 *μ*L) were then separated on 1.5% agarose gel and quantified by densitometry using Scion Image. Expression was normalized to GAPDH.

### 2.4. Coronary Angiography and Syntax Scoring

High-risk patients with CAD, as well as other patients suitable for the procedure according to the latest guidelines, underwent coronary angiography within 72 h of admission to confirm ischemic angina. The locations of coronary lesions, the number of stenosed arteries, and the degree of stenosis were recorded. Syntax scores were calculated from the angiograms using a validated web-based syntax scoring system [20, 21]. This procedure was done by two experienced cardiologists blinded to clinical data.

### 2.5. Assay of Inflammatory Cytokines

Blood samples were drawn upon admission into vacuum containers containing ethylenediaminetetraacetic acid and stored at −80°C until analysis. Plasma levels of the inflammatory cytokines interleukin-6 (IL-6), tumor necrosis factor-*α* (TNF-*α*), monocyte chemoattractant protein-1 (MCP-1), and high-sensitivity C-reactive protein (hs-CRP) were measured using enzyme-linked immunosorbent assay kits (R&D Systems, MN, USA). Measurements were collected in duplicate and averaged.

### 2.6. Statistical Analysis

Values are reported as mean ± standard deviation. Student's *t*-test or Wilcoxon matched-pairs, signed-ranks test was used to determine differences between groups Gaussian and non-Gaussian data, respectively. *χ* ^2^ test or Fisher's exact test was used to compare dichotomous data. *p* values < 0.05 were considered statistically significant. Data were analyzed in SPSS 16.0 (SPSS, Chicago, IL, USA).

## 3. Results

### 3.1. Clinical Characteristics

Enrolled patients had mean age of 58.3 years, and 62.4% were male. Details of patient characteristics are listed in Table 1. Patients with or without T2DM were not significantly different in terms of age, gender distribution, history of hypertension and hyperlipidemia, smoking status, CG-GFR, DBP, and ongoing drug therapy, which included antiplatelet drugs, beta-blockers, ACEI/ARB, statins, diuretics, and CCB. However, there were significant differences in terms of BMI, SBP, heart rate, LVEF, blood glucose, glycosylated hemoglobin, and insulin treatment.

### 3.2. SIRT1 Expression

**Figure 1:**
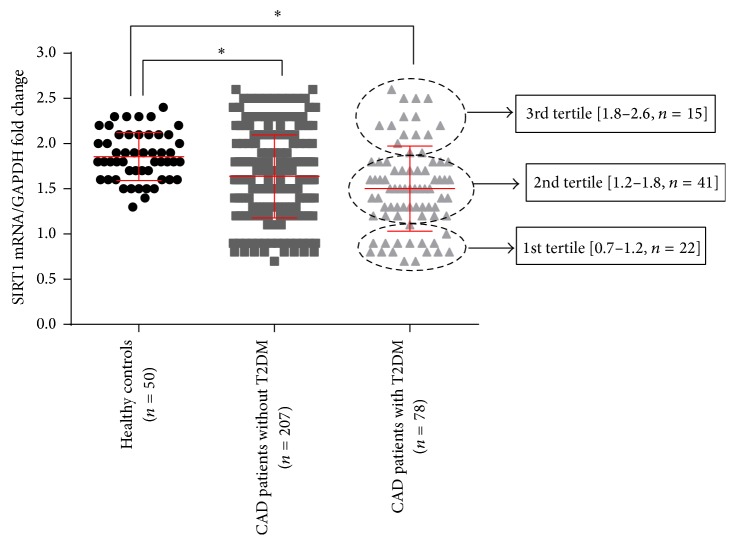
mRNA expression of SIRT1. There is no significant difference between patients with or without T2DM. In patients with T2DM, data are divided into tertiles: 1st tertile < 1.2, 2nd tertile 500 < 1.8, and 3rd tertile ≤ 2.6. ^*∗*^ *p* < 0.05.

The relative expression of SIRT1 in healthy individuals and CAD patients with or without T2DM was 1.85 ± 0.04, 1.64 ± 0.03, and 1.50 ± 0.05, respectively. Expression was significantly lower in patients with CAD and diabetes (Figure 1; *p* < 0.01). Additionally, normalized expression at the 25th percentile was 1.2 and at the 75th percentile 1.8 (Figure 1).

### 3.3. Syntax Scores

**Figure 2:**
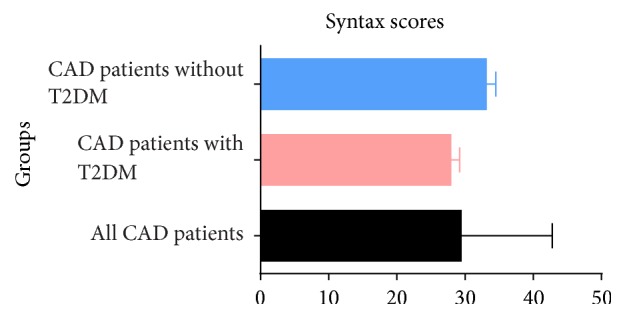
Syntax scores in three groups.

Syntax scores in all patients and in patients with or without T2DM were 29.4 ± 13.4, 33.1 ± 1.4, and 27.9 ± 1.3, respectively. There is no significant difference between diabetic and nondiabetic patients (*p* = 0.089, Figure 2). SIRT1 expression did not significantly correlate with syntax scores in CAD patients with or without T2DM (all *p* > 0.05).

### 3.4. Inflammatory Cytokines and SIRT1 Expression

**Figure 3:**
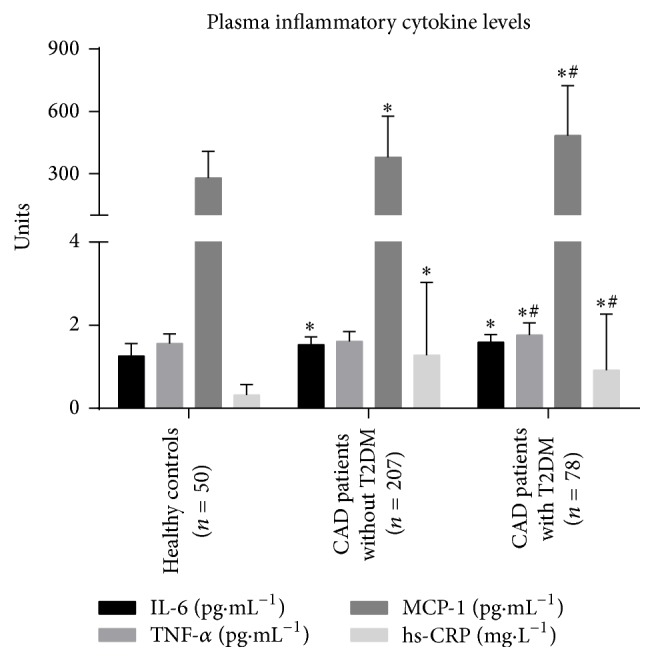
Levels of inflammatory cytokines in the plasma. TNF-*α*, MCP-1, and hs-CRP levels are significantly higher in diabetic patients than in nondiabetic patients. ^*∗*^ *p* < 0.05 versus healthy subjects; ^#^ *p* < 0.05 versus nondiabetic patients.

Circulating levels of IL-6 were 1.26 ± 0.30, 1.59 ± 0.19, and 1.53 ± 0.19 pg/mL in healthy individuals and in CAD patients with or without T2DM, respectively. The difference between patients with CAD and healthy controls was significant (*p* < 0.001), but there was no significant difference between diabetic and nondiabetic patients (*p* = 0.057, Figure 3). On the other hand, plasma TNF-*α* in diabetic patients was significantly higher (*p* < 0.001) at 1.76 ± 0.30 pg/mL than in nondiabetic patients and healthy controls, who had comparable (*p* = 0.172) TNF-*α* levels of 1.56 ± 0.23 and 1.61 ± 0.24 pg/mL, respectively (Figure 3). Additionally, plasma hs-CRP was significantly higher in nondiabetic patients than in control subjects and diabetic patients (*p* < 0.05 and *p* < 0.001, Figure 3). Hs-CRP was 0.32 ± 0.25, 1.28 ± 1.75, and 0.92 ± 1.35 mg/L in healthy, nondiabetic, and diabetic patients, respectively. Finally, plasma MCP-1 was 279.9 ± 127.9, 378.2 ± 198.8, and 483.4 ± 240.4 pg/mL in healthy individuals and nondiabetic and diabetic patients, respectively. The difference between diabetic and nondiabetic patients was significant (*p* < 0.001, Figure 3).

**Figure 4:**
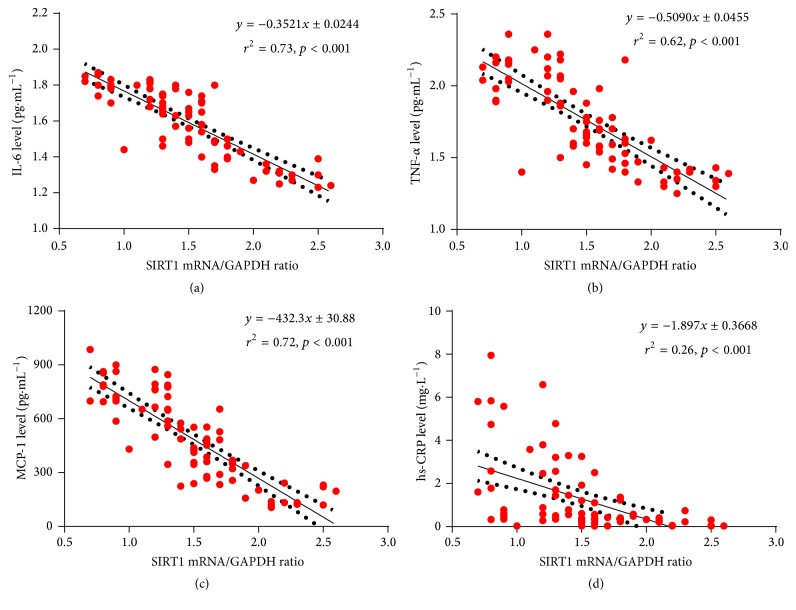
Correlation between levels of inflammatory cytokines and SIRT1 expression. SIRT1 expression was negatively correlated with plasma IL-6 (a), TNF-*α* (b), MCP-1 (c), and hs-CRP (d); *p* < 0.05.

The level of TNF-*α* was found to significantly relate to peripheral SIRT1 expression in monocytes in patients without T2DM (*r* ^2^ = 0.026; *p* = 0.0193). There were no significant correlations between SIRT1 expression and other plasma levels (IL-6, hs-CRP, and MCP-1) in this study (*p* = 0.0524, *p* = 0.3353, and *p* = 0.8814). However, SIRT1 expression was significantly correlated with levels of inflammatory cytokines in patients with T2DM (*p* < 0.05, Figures 4(a)–4(d)).

## 4. Discussion

Macrovascular complications are common in patients with T2DM who then present accelerated atherosclerotic changes in vessels [22]. Growing evidence suggests that SIRT1 can be regarded as a new therapeutic target to prevent or reverse such complications [23–25]. However, the exact relationship between SIRT1 expression and coronary arterial stenosis is poorly understood. Our data suggest that SIRT1 expression in peripheral blood mononuclear cells is not associated with the severity of coronary lesions in patients with T2DM but is associated with levels of inflammatory cytokines in the plasma. This finding may partly explain outcomes in patients with both CAD and T2DM.

Crujeiras and colleagues [26] assessed SIRT1 expression in peripheral blood mononuclear cells to investigate the effect of the sirtuin pathway on obesity therapy. de Kreutzenberg et al. [27] reported that insulin resistance and metabolic syndrome were associated with low SIRT1 expression in the same cells and that SIRT1 expression was negatively correlated with subclinical atherosclerosis. Song et al. [28] provided some evidence that SIRT1 might have a role in the pathogenesis of T2DM, and its expression in granulocytes and monocytes might indirectly reflect metabolic status in diabetic patients. Many studies have also demonstrated that SIRT1 might suppress inflammation and thus be used to manage inflammatory disorders [29–31]. In accordance with these results, our data link SIRT1 to inflammatory biomarkers in patients with CAD and T2DM.

Sirtuins (SIRT1–SIRT7) are a family of nicotinamide adenine dinucleotide-dependent enzymes. SIRT1, an important member of this family, is involved in a wide range of physiological and pathological processes [32]. For example, SIRT1 promotes reverse cholesterol transport in macrophages by regulating the oxidized low-density lipoprotein receptor-1 (LOX-1) and the liver X-receptor (LXR) and by inhibiting formation of foam cells [12, 15]. On the other hand, SIRT1 overexpression or activation suppresses expression of adhesion molecules in endothelial cells through the NF-*Κ*b pathway and thereby improves endothelial function [16]. Gorenne et al. [33] also found that SIRT1 expression in vascular smooth muscle cells protects against DNA damage, medial degeneration, and atherosclerosis. Indeed, SIRT1 activity may be involved in the development of atherosclerosis and is thus a promising target for novel drugs against CVD and related diseases [34].

Furthermore, SIRT1 has been implicated in glucose homeostasis and lipid metabolism in adipose tissue, liver, pancreas, and skeletal muscle [35]. Moreover, SIRT1 activation reduced diabetes symptoms and insulin secretion and delayed the onset of insulin resistance in diabetic rats [36]. On the other hand, Lagouge et al. [37] reported that the SIRT1 activator resveratrol enhanced insulin sensitivity* in vitro* and attenuated high fat diet-induced obesity and insulin resistance* in vivo*. This compound has also been shown to not only improve glucose metabolism and vascular function in patients with prediabetes but also improve left ventricle diastolic function and endothelial function in patients with CAD [38, 39]. Similarly, highly selective SIRT1-activating compounds improved insulin sensitivity, lowered plasma glucose, and increased mitochondrial capacity in diet-induced obese and genetically obese mice [40]. Finally, the SIRT1 activator SRT2104 was also shown to significantly affect serum cholesterol, LDL, and triglycerides [41].

However, these results remain controversial. Poulsen et al. [42] found that resveratrol did not affect blood pressure, resting energy expenditure, oxidation of lipid, ectopic or visceral fat, and inflammatory and metabolic biomarkers. Similarly, Semba et al. [43] reported that total urinary resveratrol metabolites in adults were not associated with inflammatory markers and cardiovascular disease or predictive of all-cause mortality. Thus, large-scale clinical studies may be necessary to resolve these contradictory results.

Although our sample size was relatively large, there were several limitations. While we did not observe a relationship between SIRT1 expression and the severity of coronary lesions in patients with both CAD and T2DM, the exact role of SIRT1 in atherosclerosis is more profound than most studies reported. Moreover, the contribution of SIRT1 to glucolipid metabolism and inflammatory signaling is not well characterized. Whether SIRT1 prevents atherosclerosis in diabetic patients remains to be seen.

## 5. Conclusions

In summary, the data demonstrate that SIRT1 expression in peripheral blood mononuclear cells is not associated with the severity of coronary lesions in patients with CAD and T2DM. However, SIRT1 mRNA expression is significantly correlated with IL-6, TNF-*α*, MCP-1, and hs-CRP levels. Further research is needed to characterize the effects of SIRT1 on the development of atherosclerotic lesions.

## Figures and Tables

**Table 1:** Demographic and baseline clinical characteristics.

	Healthy controls	Patients with CAD	Patients with CAD and T2DM
*n*	50	207	78
Age (y)	37.4 ± 1.4	58.3 ± 0.6	58.4 ± 1.1
Gender (male, %)	31 (62%)	124 (59.9%)	54 (69.2%)
Hypertension, *n* (%)	0 (0)	114 (55.1%)	44 (56.4%)
Hyperlipidemia, *n* (%)	0 (0)	120 (58.0%)	58 (66.7%)
Smoking, *n* (%)	0 (0)	122 (58.9%)	47 (60.2%)
BMI (kg/m^2^)	24.0 ± 0.4^*∗*^	24.1 ± 0.2^#^	25.2 ± 0.3
CG-GFR (mL/min)	92.1 ± 0.9^*∗*^	88.1 ± 0.7	85.4 ± 1.3
SBP (mmHg)	128.4 ± 1.8^*∗*^	131.3 ± 1.1^*∗*^	136.2 ± 2.3
DBP (mmHg)	73.5 ± 1.3	74.6 ± 0.7	74.8 ± 1.2
Heart rate (beats/min)	73.6 ± 1.3^*∗*^	74.4 ± 0.8^*∗*^	77.5 ± 1.4
LVEF (%)	63.2 ± 0.9^#^	61.8 ± 0.5^#^	58.8 ± 0.9
Fasting blood glucose (mmol/L)	5.3 ± 0.3^#^	5.5 ± 0.4^#^	7.2 ± 0.3
HbA1C (%)	5.2 ± 0.3^*∗*^	5.4 ± 0.2^*∗*^	6.9 ± 0.4
Duration of diabetes (years)	0	0	3.4 ± 1.1
*Drug therapy*			
Antiplatelet drugs, *n* (%)	0 (0)	133 (64.3%)	51 (65.4%)
Beta-blocker, *n* (%)	0 (0)	78 (37.7%)	30 (38.5%)
ACEI/ARB, *n* (%)	0 (0)	94 (45.4%)	36 (46.2%)
Statins, *n* (%)	0 (0)	127 (61.4%)	42 (53.8%)
Diuretics, *n* (%)	0 (0)	26 (12.6%)	9 (11.5%)
CCB, *n* (%)	0 (0)	103 (49.8%)	38 (48.7%)
Insulin, *n* (%)	0 (0)	0 (0)^#^	17 (21.8%)

BMI: body mass index; CG-GFR: Cockcroft-Gault glomerular filtration rate; SBP: systolic blood pressure; DBP: diastolic blood pressure; LVEF: left ventricular ejection fraction; HbA1C: glycosylated hemoglobin; IL-2: interleukin-2; hs-CRP: high-sensitivity C-reactive protein; TNF-*α*: tumor necrosis factor-*α*; MCP-1: monocyte chemotactic protein-1; ACEI: angiotensin converting enzyme inhibitor; ARB: angiotensin II receptor blocker; CCB: calcium channel blocker. ^*∗*^ *p* < 0.05 versus CAD patients with diabetes; ^#^ *p* < 0.01 versus CAD patients with diabetes.
